# Staffing in postnatal units: is it adequate for the provision of quality care? Staff perspectives from a state-wide review of postnatal care in Victoria, Australia

**DOI:** 10.1186/1472-6963-6-83

**Published:** 2006-07-04

**Authors:** Della A Forster, Helen L McLachlan, Jane Yelland, Jo Rayner, Judith Lumley, Mary-Ann Davey

**Affiliations:** 1 Mother and Child Health Research, La Trobe University, 251 Faraday St, Carlton 3053, Australia; 2 Clinical School of Midwifery and Neonatal Nursing Studies, La Trobe University, Bundoora, 3083, Australia

## Abstract

**Background:**

State-wide surveys of recent mothers conducted over the past decade in Victoria, one state of Australia, have identified that women are consistently less satisfied with the care they received in hospital following birth compared with other aspects of maternity care. Little is known of caregivers' perspectives on the provision ofhospital postnatal care: how care is organised and provided in different hospitals; what constrains the provision of postnatal care (apart from funding) and what initiatives are being undertaken to improve service delivery. A state-widereview of organisational structures and processes in relation to the provision of hospital postnatal care in Victoria was undertaken. This paper focuses on the impact of staffing issues on the provision of quality postnatal care from the perspective of care providers.

**Methods:**

A study of care providers from Victorian public hospitals that provide maternity services was undertaken. Datawere collected in two stages. Stage one: a structured questionnaire was sent to all public hospitals in Victoria that provided postnatal care (n = 73), exploring the structure and organisation of care (e.g. staffing, routine observations, policy framework and discharge planning). Stage two: 14 maternity units were selected and invited to participate in a more in-depth exploration of postnatal care. Thirty-eight key informant interviews were undertaken with midwives (including unit managers, associate unit managers and clinical midwives) and a medical practitioner from eachselected hospital.

**Results:**

Staffing was highlighted as a major factor impacting on the provision of quality postnatal care. There were significant issues associated with inadequate staff/patient ratios; staffing mix; patient mix; prioritisation of birth suites over postnatal units; and the use of non-permanent staff. Forty-three percent of hospitals reported having only midwives (i.e. no non-midwives) providing postnatal care. Staffing issues impact on hospitals' ability to provide continuity of care. Recruitment and retention of midwives are significant issues, particularly in rural areas.

**Conclusion:**

Staffing in postnatal wards is a challenging issue, and varies with hospital locality and model of care. Staff/patient ratios and recruitment of midwives in rural areas are the two areas that appear to have the greatest negative impact on staffing adequacy and provision of quality care. Future research on postnatal care provision should include consideration of any impact on staff and staffing.

## Background

Having a baby is the most common reason for hospitalisation in Australia, and each year around 63,000 women give birth in Victoria. A decade of Victorian research has found low levels of satisfaction with the hospital stay following birth. Women are less likely to be satisfied with postnatal care than antenatal and intrapartum care, and only 51% of the women participating in a state-wide Survey of Recent Mothers in 2000 (SRM 2000) rated their postnatal care as 'very good' [1].

Other studies from Australia and internationally have reported similar findings in relation to women's satisfaction with care – that women rate postnatal hospital care less favourably than the other episodes of maternity care [2-4] or that postnatal care could have been improved [5].

In SRM 2000, the factors which had the strongest associations with less positive ratings of postnatal care were those which had to do with women's experiences of specific aspects of care: the sensitivity of caregivers; extent to which anxieties and concerns were taken seriously; how rushed caregivers seemed; the helpfulness of advice and support, and whether help and advice was offered at all [1]. These findings are similar to other reports [5,6]. Given that these factors all relate to interactions between new mothers and care providers, it is important to explore the issue of staff and staffing in relation to postnatal care provision.

Other factors associated with women's experience of postnatal care in SRM 2000 were locality and accommodation status. Women in *rural *areas were more likely to rate their postnatal care as 'very good' (56%) than women in metropolitan areas (49%) [7]. Of women who had a *public *booking for maternity care, 53% of women giving birth in rural Victoria considered postnatal care 'very good', which was significantly higher than the 42% of women giving birth as public patients in metropolitan hospitals who rated their care 'very good'. Women with *private *bookings for maternity care were more likely to rate postnatal care as 'very good' overall, with 66% of women receiving care in rural settings rating postnatal hospital care as 'very good' and 62% of the metropolitan respondents. Of the births in Victoria, around 42,300 women give birth and receive postnatal care in public hospitals (Mary-Ann Davey, Victorian Perinatal Data Collection Unit, personal communication); that is, 68% of all confinements in Victoria are in public hospitals. Approximately 5% of women choosing private maternity care also give birth and receive care in public hospitals. Issues related to staffing may be affected by both locality and accommodation status.

Our recently completed state-wide review of **p**ostnatal **in**-hospital **c**are (PinC) explored postnatal care from the care provider's perspective [8]. There was great diversity in the provision of postnatal care across Victoria with differences in models of care, staffing arrangements, physical facilities and routine practices. Overall, care providers were enthusiastic about postnatal care, and committed to ensuring this care was of high quality. However, there was a strong sense that postnatal care provision is considered a lower priority, or 'poor cousin' compared with other episodes of maternity care. The major barriers identified to the provision of high quality care were the busyness of the postnatal units; the inadequacy of staff-patient ratios; staff shortages in rural areas; the priority given to other episodes of care; and the lack of opportunity for women to rest. Two of the eight recommendations arising from the PinC review relate to issues about staffing: that staff-patient ratios in postnatal units be reviewed and periodically monitored by the Australian Nursing Federation (ANF), the Australian College of Midwives Incorporated (ACMI) and the Victorian Department of Human Services (DHS); and that DHS give priority to recruitment of midwives in rural areas [8].

The aim of this paper is to describe staffing and related issues in the provision of postnatal care in public hospitals in Victoria from the caregivers' perspective; and to explore what impact (if any) providers believe staffing issues have on their ability to provide high quality care to women.

### The regulation of staffing in postnatal care in Victoria, Australia

In Victoria nurses and midwives are registered to practise by the Nurses Board of Victoria. Division 1 nurses are licensed to practise nursing in the field/s in which they are registered, and to practise as part of a team, independently and inter-dependently in accordance with professional standards [9]. To practise as a midwife, Division 1 nurses are required to be endorsed as a midwife by the Board, and the title 'midwife' is a protected title [9]. Other nursing staff able to provide maternity care under the direction of a registered nurse or midwife are Division 2 nurses (referred to as Enrolled Nurses in some jurisdictions), who have no formal preparation in maternity care; and Mothercraft nurses, who have basic education in providing care for babies and young infants, although in Victoria there is no option for further new registrations as a mothercraft nurse, that is, the register is closed [9]. There are currently four pathways of midwifery education: a one year postgraduate diploma for registered nurses (Graduate Diploma of Advanced Nursing (Midwifery)); a one year postgraduate diploma for registered nurses where part of the required clinical hours are undertaken as a paid hospital employee (Graduate Diploma of Advanced Nursing (Midwifery); a three year undergraduate degree (Bachelor of Midwifery); and a four year undergraduate double degree (Bachelor of Midwifery and Bachelor of Nursing).

A full-time workload as a midwife or nurse is 38 hours per week. Given that hospital postnatal care is provided 24 hours per day for seven days a week, it requires 5.6 people working equivalent to full-time (EFT) to provide one midwife for three shifts each day on a ward, inclusive of annual leave. The ANF, the national professional body for nurses, has set nurse/patient ratios that hospitals in the Victorian public sector have agreed to follow. These ratios were introduced in 2000 to address untenable workload problems, and severe staffing shortages, with the aim of establishing a sustainable and efficient health care system in Victoria.[10] On postnatal wards these ratios are one to five on a day shift (one staff member to five women *exclusive *of the baby, i.e. mother and baby are treated as one individual), one to six on an afternoon shift and one to eight on the night shift [11]. Midwives are not involved in general cleaning tasks, routine bed cleaning or food preparation/distribution in Victoria.

## Methods

The PinC review aimed to explore the structure and organisation of hospital postnatal care and identify those aspects of care impacting on women's experiences and maternal and infant health. The review included two methods of data collection: a survey of all public maternity hospitals in Victoria, Australia regarding current guidelines and procedures governing care, followed by detailed interviews with managers and care providers. The two methods used are described separately below.

### Survey of public hospitals

Public maternity care in Victoria is provided by four main hospital types: tertiary maternity hospitals (major referral centres, with neonatal intensive care facilities); metropolitan non-tertiary hospitals; large regional hospitals (non-metropolitan units servicing a specific rural/regional area, with special care neonatal facilities, but not the facility to care for babies needing ventilator support); and smaller rural hospitals.

#### Participants

All public maternity care providers in the state were identified by DHS (n = 73). Each hospital was contacted by telephone to ascertain the name and contact details of the appropriate person to whom the questionnaire should be sent, for example the Director of Nursing or a Midwifery Unit Manager. Further information was obtained at the time of the initial telephone contact regarding the eligibility of hospitals for participation, as some were no longer providing any maternity services.

#### Data collection

A survey instrument was developed by the PinC team, incorporating suggestions and feedback from a reference group convened for the project. Piloting was undertaken initially with personal contacts, such as colleagues who worked in postnatal wards of (private) hospitals that were ineligible for participation. The survey was refined and re-piloted with unit managers of postnatal wards of ineligible hospitals (rural and metropolitan). Feedback from these processes resulted in a number of modifications and additions prior to the final version of the questionnaire.

The survey comprised both open and closed-ended questions on a range of topics related to the provision of maternity care at the particular hospital, including: hospital location; number of births (if applicable); models of care; guidelines and protocols; and initiatives in postnatal care. One section was dedicated to questions around staffing. Question areas included: staff numbers providing postnatal care; part time/full time staff complements; average number of staff per shift; midwifery student types and numbers; type of professional staff providing postnatal care; staff ratios; use of casual and bank staff; and adequacy of staffing. Hospitals were each assigned a unique identifier (ID). Each hospital received a courtesy call a week after the survey was sent, to ensure they had received it. Written and telephone reminders were undertaken after three and five weeks respectively.

#### Data analysis

Data were entered onto an Access database [12] and analysed using STATA [13]. Where questions had pre-coded response options, analysis was largely undertaken using descriptive statistics, including frequencies, proportions and medians (few of the variables were normally distributed). Open-ended responses were coded and collapsed into themes. In many instances, data were stratified by hospital size and location. Direct quotes from the survey and key informant interviews are used in this paper. Quotes from the survey are identified by '(survey-hospital ID, hospital category/location)'. Victoria has five rural and four metropolitan health regions, and birth outcomes are often considered in this context. Many of the results in this paper are sub-divided by hospital location: metropolitan (tertiary and non-tertiary), and non-metropolitan (regional and rural).

### Key informant interviews

#### Participants

Key informant interviews were incorporated into the PinC review with the aim of providing a more detailed understanding of the views and experiences of clinicians and managers involved in postnatal care. We planned to conduct 52 interviews with key informants from 14 public maternity units across Victoria. Two sampling strategies (purposive and random) were employed to select participating hospitals with the aim of obtaining a wide range of views to reflect the care of postnatal units across the state. All tertiary hospitals and a sample of other hospitals were included to ensure we heard from care providers working in diverse settings. Hospitals were stratified by number of births per year and location. Participants were selected from: all tertiary hospitals (three); and other hospitals randomly selected (by a person independent of the research team) in a number of categories – four (of eight) metropolitan non-tertiary hospitals; three (of six) regional (Base) hospitals; and four (of 25) hospitals from rural areas with greater than 100 births per year.

Additional information was requested from hospitals selected to participate in key informant interviews, including contact details of a maternity unit manager, an associate unit manager, a clinical midwife and a medical practitioner who had agreed to be contacted by the PinC team to participate in an interview. We asked for only three nominees from the four rural hospitals because of fewer staff numbers. Unique identifiers (IDs) were assigned to each key informant, and were related to the hospital IDs. Written consent was obtained from key informants prior to commencement of each interview.

#### Data collection

The interview schedule was a semi-structured questionnaire, and was developed using themes emerging from the hospital survey, as well as areas identified in the literature. Feedback from reference group members was also incorporated. Questions relevant to this analysis focused on how care was organised in terms of staffing, for example: adequacy of staffing; skill mix; and the influence or impact of part time or casual staff. Respondents were also asked to comment on the busyness of postnatal wards, a common theme arising from the survey. We planned to conduct key informant interviews face-to-face and to audio-tape them.

The key informant interview schedule was piloted in the first instance with colleagues (including audio-taping); these included a former postnatal unit manager, a former associate postnatal manager and two midwives who worked on postnatal wards. Following these pilot interviews, the *content *of the questions remained the same but the questions were reworded and the structure changed to make the questions clearer and more precise. Following these changes the schedule was piloted on two more occasions with currently practicing midwives.

#### Data analysis

Key informant interviews were transcribed verbatim onto word processing software. All potentially identifying information about individual hospitals was removed. The transcripts were checked against the audio-tapes and then coded thematically. Basic themes and categories (previously identified due to the semi-structured nature of the interviews) were fully explored in each transcript, and coded first into sub-themes, then collapsed under organisational theme headings. Coding was cross-checked for inter-coder reliability. Direct quotes of comments from the key informant interviews are identified by '(KI-hospital ID- KI ID, hospital category/location)', and were selected to reflect the major emergent themes.

Research ethics approval was obtained from the DHS and La Trobe University Research Ethics Committees. Further ethics approval was gained from individual health services as necessary (ten hospitals).

## Results

### Who responded?

All eligible hospitals were sent a PinC questionnaire in April/May 2004. Four of the 73 hospitals had not provided postnatal care for 12 months or more and were considered ineligible. Sixty-six of 69 (96%) eligible hospitals responded. Of the 66 hospitals completing the survey, 43 (65%) were located in rural areas; ten (15%) in regional areas; ten (15%) were metropolitan non-tertiary; and three (4.6%) were tertiary hospitals located in the metropolitan area. Six hospitals provided postnatal care only (and no antenatal or intrapartum care). One metropolitan non-tertiary hospital and two rural hospitals did not respond. A high proportion (67%) of the hospitals had fewer than 500 births in 2002, with 33% having fewer than 100 births for the year (Table 1).

Key informant interviews were conducted between August and October 2004. Thirty-eight of an anticipated 52 interviews were conducted. In some instances participating hospitals nominated fewer than the requested number of key informants and on occasions key informants were unavailable for interview. After 38 interviews we reviewed the need to seek replacements and decided (following initial analysis) we had reached data saturation, that is, no new themes or views were emerging; there was a consistency in respondent views. All but two interviews were conducted face-to-face; telephone interviews were conducted with the remaining two key-informants.

The key informants represented all hospital locations, sizes and clinical areas and had a great deal of experience in maternity service provision. There were 11 midwifery and/or nursing unit managers, 14 associate unit managers, eight clinical midwives and five medical practitioners. Nine key informants came from the tertiary hospitals, ten from metropolitan, eleven from regional and eight from rural hospitals. Thirty-four key informants had worked in maternity care for more than ten years, thirty worked full-time and twenty-four midwives combined clinical care with a managerial role. All midwives were female. Of the five medical practitioners interviewed (three obstetricians and two GP/obstetricians) two were male and three female.

### Who staffs postnatal wards in Victoria?

Excluding the two rural and one metropolitan non-tertiary hospital that did not respond to the survey 2,000 midwives were employed in public hospitals in Victoria to provide postnatal care at the time the survey was completed. This included midwives who worked only in postnatal wards as well as midwives who rotated through postnatal wards and other areas. It did not include midwives who were casual staff, or who were employed through a nursing agency. Around 63% of the 63,000 births in Victoria each year are to women are booked as public patients,[14] which means that 2,000 midwives are employed by hospitals to provide postnatal care for approximately 42,300 women. These figures do not take into account the part-time/full-time status of midwives, the use of staff other than midwives who provide in-hospital postnatal care, or the use of casual and agency staff. However, they do give some indication of the absolute numbers of midwives needed to provide postnatal care in its current form.

The 2000 midwives employed by public hospitals (at the time of the survey) to provide postnatal care equated to 1,025 full-time equivalent (EFT) staff. Only 28% of midwives were employed to work *exclusively *in the postnatal area at the time of the survey. Except in the three tertiary hospitals, midwives were almost all employed on a part-time basis, and rotated through postnatal and other maternity areas, as well as providing non-maternity care in many of the smaller hospitals.

Many hospitals reported having staff other than midwives regularly providing postnatal care to women (Table 2), with only 43% of hospitals reporting having *just *midwives providing hospital postnatal care. Twenty percent of hospitals employed mothercraft nurses, and this was throughout all hospital categories. Thirty-seven percent employed Division 2 nurses. This was again throughout all hospital categories. Nine percent had Division 1 nurses not endorsed as midwives regularly providing postnatal care, however this was confined to rural and regional hospitals. Given that only 65% (28/43) of the rural hospitals reported on this question, these figures are likely to under-represent the true situation.

Some key informants indicated that whilst these arrangements often existed to fill a need, midwives may not always see the situation as ideal. When non-midwifery staff provide postnatal care the care tends to be fragmented because they are not qualified to carry out the full range of activities:

There's one mothercraft nurse, and... she's helping with their [the midwives] load, but then they feel like they're not totally fulfilling their role of the mother and baby unit together (KI-1020-1, regional)

Sometimes we might get a Div 2 nurse possibly come around for a while to do some obs and make beds, but really it's not that advantageous because when you've got problems with ... breastfeeding and all that stuff ... you really need another midwife (KI-1036-2, rural)

### Casual, bank and agency staff

As well as permanent midwives and non-midwives providing postnatal care, both full-time and part-time, most hospitals also reported using casual staff, both bank and agency. Bank staff are those staff (midwives in this case) employed by the hospital on a casual basis, who usually work in a variety of areas, but who are called on to work only as needed. That is, they are not guaranteed work by the hospital, and conversely, they are not required to work each week on a rostered basis as would be the case if they were employed as permanent staff. Agency staff work in the same way, but are employed by private 'agencies', not by individual hospitals. A key informant talked about this staffing mix in both positive and negative terms:

We do have a vast mixture of personnel looking after the mothers. We have student midwives, our core staff, bank and agency, and I think that has a very good impact on the quality of care for mothers... but because the majority of our staff are not full-timers it is very difficult to give continuity of care. I would say out of my 26 staff... there would be two, maybe three of us that work full-time (KI-1046-1, tertiary)

Overall, 66% (43/66) of hospitals reported that they had a nurse/midwife casual bank of staff that could be called on to fill gaps in staffing. Some *rural *hospitals did not have this option, with only 47% (20/43) reporting having a nurse/midwife casual bank. The key informant interviews give some idea of the issues with bank staff in rural areas; mainly that there is a general lack of availability of staff to employ in this capacity:

We keep getting told there are lots of bank staff available, but the reality is there are not, and many, many times the person in charge... will make 20 to 30 phone calls looking for staff ... there's just nobody... particularly on night duty which is... really difficult (KI-1020-3, regional)

We've only got... a handful of casual midwives, most of us work part-time... there are midwives we can call upon but otherwise it's just [a] staff member getting called, saying 'we're busy can you come in'. What often will happen is if we do have somebody maybe who's in labour and we've got a busy ward with 'postnates', the second midwife in [the general ward area] will come around to work here and they will replace her with a Division 1 nurse (KI-1036-2, rural)

Yes, we have a bank... [but] most of our bank unfortunately are ... only available at the weekend (KI-1012-1, regional)

A number of key informants indicated that they either did not have a casual bank, or that the number of staff available to work bank was very low. A solution was to ask part-time staff to do extra shifts, or to 'juggle' staffing to suit the needs:

...we call often on the part-time midwives to help us when we're busy... we've got some casual bank midwives but we have trouble getting them to cover all shifts (KI-1020-1, regional)

Only 29% of hospitals (19/66) who responded to the survey reported that they used the services of a private nursing agency. A number of key informants also indicated that they try to avoid the use of agency staff when possible. This is likely to be a result of the Victorian State Government initiative in 2002, when due to the escalating costs of private agency nursing staff, the Government encouraged hospitals to develop (or increase the use of) their own casual banks of nursing and midwifery staff, and recommended that hospitals should limit the use private agency staff to unplanned absences and exceptional circumstances [15]. Of the hospitals that reported using agency staff, 78% had used *no *agency staff to provide postnatal care in the month designated in the survey. When agency staff *are *used, it appears to be a relatively common practice that they are sent to postnatal areas, with postnatal staff then sent to (for example) birth suite:

...with delivery suite, if... there's sick leave or whatever and they need more staff, it is taken from the ward, and... that annoys us at times, but there's no other way around it..., and we tend to then... find some bank or agency or whatever... in other words they don't tend to have agency in there, we tend to have them out here [in postnatal], which I think is reasonable (KI-1011-3, metropolitan non-tertiary)

Some hospitals, particularly in rural settings, don't use agency staff because there "*...are no agency nurses really*" (KI-1066-4, rural).

In many hospitals there are now guidelines about use of agency staff. Many nursing agencies do not have insurance to cover their staff working in birth suite or antenatal areas. This has resulted in the practice of using agency staff in postnatal wards rather than other maternity areas, as highlighted by one respondent:

We can't have agency staff on an antenatal ward, they're not covered for their insurance, and we can't have them in labour ward because they're not covered by insurance, so it means that they go to a postnatal ward because that's what they're covered for. Now that's great because we can get someone, but you often feel like the poor cousin (KI-1053-3, tertiary)

So the use of agency staff in particular, appears to have its own impact on postnatal wards. If staff *are *needed in other areas of maternity, postnatal core staff may be sent to those areas, and agency staff used to cover the gap in postnatal. Some key informants reported that agency staff may be given a lighter load, adding to the load on those permanent staff who remain on the ward:

I always try to give them a lighter load so I don't put too much stress on them because they're not familiar with the...work environment, so I never tend to give them a heavy load...(KI-1046-2, tertiary)

Two key informants made comments on the fact that agency staff may or may not be satisfactory, or up to the standard they expect:

Occasionally we have to use agency staff, and some of them are fantastic and some aren't (KI-1053-3, tertiary)

[Some agency staff] don't like coming here, which is pretty sad... some of it is staffing issues, some of it is the hard work they have to do and some of it is the expectation that we have of them, and some of it sometimes [is] that they're not capable of fulfilling the role and some of these people haven't got good basic nursing skills let alone midwifery skill (KI-1048-1, tertiary)

In turn, agency staff may be reluctant to return to hospitals or wards that are regularly understaffed, as they are only called in when it is absolutely necessary.

### Student midwives

The hospital survey showed that student midwives had placements at all tertiary hospitals, 80% of metropolitan non-tertiary hospitals, all regional hospitals and in one-third of rural hospitals. Hospitals take student midwives in various capacities including undergraduate, a postgraduate paid model and a postgraduate unpaid model. Sixty percent of hospitals reported having students only in a supernumerary capacity and 29% reported having students who worked a mixture of supernumerary and paid days. Eleven percent of hospitals reported they did not offer supernumerary positions for student midwives.

The patient load students are expected to take varied considerably between hospitals. For example, one third of all hospitals expected students to take a full patient load, despite their student status. One quarter of hospitals expected students to take a 75% patient load and two hospitals expected students to take a 50% load. In four hospitals (13%), students worked with midwives *at all times *without any patient load of their own.

Some of the comments from the key informant interviews demonstrate the complexities associated with working with the different student midwives, and being aware of the students' varying learning needs. Student midwives may help with staffing levels in some instances, but at other times may increase the workload for midwives on postnatal wards:

...if you've got patients plus a student to care for, you're full on (KI-1011-2, metropolitan)

In some cases respondents reported working with a wide range of midwifery students as well as students from other disciplines. This is likely to increase the hospitals' difficulty in understanding the various clinical placement requirements of the relevant institutions:

[We have] lots of students. We have midwifery students...doing their double degree ... and we also have them doing the post grad year... we've had ...students ... doing that direct entry course, we have a Year 5 medical student we have ... paramedics ... occasionally, I've had a year four physio student occasionally... they want to come and observe a birth and postnatal care, we have work experience students from Year 10 to Year 12 at times requesting to observe what a midwife does, we also have two graduate midwives doing a graduate midwife program over 12 months and they're .84 EFT (KI-1020-1, regional)

### Factors that affect staffing adequacy

Seventeen percent of survey respondents (11/65) said that they *did not *consider their postnatal unit to be adequately staffed, with a further 8% (5/65) undecided; it is likely that in many cases the surveys were completed by a staff member in a management position, and that their perception of staffing adequacy may differ from that of a clinical midwife.

Four key areas were identified by the survey respondents as impacting on the adequacy of staffing on postnatal wards: staffing mix/numbers; patient mix; the impact of staff leave (both planned and unplanned); and staff/patient ratios.

#### Staffing mix/numbers

Concerns were raised regarding staff other than midwives caring for women during the postnatal hospital stay:

I feel when we have postnatal patients in that we...require a second Div 1 nurse as our unit is staffed by one Div 1 (midwife) and one Div 2. The Div 1 gets busier with a postnatal patient in the ward (survey-1001, rural)

A mothercraft nurse is employed in postnatal and counted in staff/patient ratios (survey-1035, metropolitan non-tertiary)

A midwife is rostered on at all 3 hospitals [in the health service] on most shifts but at times a midwife is on call instead. That means a postnatal patient will at times not have a midwife on site – she may be called on if necessary but often this does not happen (survey-1024, rural)

[There is] not always a midwife available for 24 hr cover (survey-1032, rural)

Staffing adequacy also varied depending on the skill mix of staff on at the time, for example:

There is variation in midwives skills, which can increase the workload for more experienced... midwives (survey-1040, rural)

Eighty-five percent of respondents noted that the birth suite was prioritised over the postnatal ward during busy periods. While respondents were accepting that this was appropriate given the importance and unpredictability of intrapartum care, it still impacted on postnatal care. The movement of postnatal staff to other areas was reflected in comments on staffing adequacy:

When birth suite is very busy the postnatal area may not be adequately staffed (survey-1020, regional)

Depends on what is happening in other areas of the hospital...if A & E [accident and emergency] is busy, this is not [normally] staffed so staff from ward attend A & E, this has an enormous impact on the rest of the ward including the maternity patients (survey-1055, rural)

In Victoria, there is a directive from DHS that all women be offered at least one home visit by a midwife (following discharge from hospital), and that women with special needs be offered two or more home visits [16]. Different hospitals have different arrangements regarding provision of this care. In the survey, 92% of hospitals reported that their midwives provided the domiciliary care to women who gave birth in their hospital. Of these, 23/61 (38%) had a separately staffed domiciliary service, with the remainder reporting that a range of staff midwives provided the care. One key informant suggested that with domiciliary care they are:

...getting busier and busier...more and more staff are taken from the ward [leaving the ward] a bit light on (KI-1011-3, metropolitan)

If the postnatal area is quiet, staff may also be moved, but this may in turn leave no opportunity for *"quality activities [or to] just catch up" *(KI-1012-1, regional).

#### Patient mix

An issue regarding staffing adequacy and patient mix was the increased care/workload needs in postnatal wards today compared with previously. Quotes that illustrated these views included:

Workload on night duty is unmanageable with 3 staff... [the]... emotional needs of women and ongoing breastfeeding support. AM [morning] shift requires extra member of staff to adequately educate women & perform discharge paperwork. Paediatricians are wanting to admit more babies to the postnatal floor, requiring [three hourly] feeds and blood glucose monitoring which is not feasible to give appropriate care with current staffing levels (survey-1070, regional)

... activity can be high due to increased number of [caesareans] and [complex] social needs of patients (survey-1053, metropolitan non-tertiary)

In 2/10 (20%) metropolitan non-tertiary, 5/10 (50%) regional and 30/43 (70%) rural hospitals, non-maternity patients are accommodated in a ward with women who have recently given birth, although only one hospital reported regularly accommodating the two in the same room. This mix of patients appeared to increase the workload of midwives, particularly in the rural setting:

Often we have to look after general patients as well, so it can become very busy and you can get pulled away to do other things e.g. admissions and help in A & E (survey-1049, rural)

It is difficult to balance the needs of midwifery clients when they are nursed in [an] area that has [competing] high needs, [such as] medical aged care patients (survey- 1010, rural)

#### The impact of staff leave

In some instances staffing was considered adequate in general, but staff leave had the potential to have quite an impact due to the difficulties of replacing staff. This was particularly relevant to rural areas:

[Staffing is adequate] unless we cannot replace sick leave (survey-1047, regional)

[Staffing is adequate] when midwives are available with no sick/holiday leave problems or workload issues (survey-1044, rural)

Replacement of sick leave by midwives can be problematic (survey-1020, regional)

#### Staff-patient ratios

The majority of survey respondents reported using the ANF ratios as the basis for their staffing. Three hospitals (of 65) reported that their ratios were based on patient acuity. Of hospitals that staffed their postnatal wards according to ANF ratios, 79% reported that the ratios were met at all times. Thirteen percent reported ratios were not met 1–5 shifts per week and 9% said that the ratios were not met 6–10 shifts per week.

However, many respondents questioned whether these ratios are adequate, particularly given the fact that although a ratio may be one to five in the morning, one to six in the afternoon and one to eight overnight, this usually *also *includes at least five (six, or eight) babies. In addition to this, babies with more complex needs, or of higher acuity, such as those requiring phototherapy, vaccinations and intravenous antibiotics are now more likely to be accommodated on the postnatal ward rather than the special care nursery. At the same time, the number of women who have had operative births, particularly caesarean section has greatly increased, decreasing the proportion of women able to provide care for their babies themselves. The issue of the ratios was raised on a number of occasions.

ANF ratios don't take into account mother and baby...ratio 1:5 is in fact 1:10. Babies often require obs, GBS [swabs] etc (survey-1029, metropolitan non-tertiary)

In busy periods [the ratios] ...are insufficient with workloads, especially with early post [caesarean] patients & breastfeeding problems, let alone [to] complete the necessary paperwork (survey-1012, regional)

ANF ratios do not meet postnatal needs, especially LUSCS [caesarean sections] (survey-1062, metropolitan non-tertiary)

### The impact of staffing on the quality of postnatal care

The key informant interviews gave some insight into the potential negative impacts arising in relation to staffing issues and the provision of quality postnatal care. The broad themes that arose were adequacy of staff/patient ratios, staff stress and issues around continuity of care versus core staffing.

#### Core staffing versus continuity models

Different views emerged regarding the place for more continuity of care models, with tensions between the views; some felt that core staffing enhanced the quality of care, whereas some viewed team care or rotating staff models as better for women. Three regional key informants specifically stated that a level of core staff in the postnatal area was desirable, for example to:

...specialise in the area [and] really know what's happening (KI-1012-3, regional)

and *... [someone to] be in charge of [and] facilitate postnatal care (KI-1012-4, regional)*

Others raised issues where the loss of core staff and the increase in rotating midwives or teams had caused problems. In two units (one tertiary and one regional) where most staff were part time and few were core, it was difficult to provide women in the postnatal unit with any continuity as there were different staff working in postnatal each day. In two other metropolitan units where midwives worked in all areas, the perception was that the more skilled or experienced staff tended to work in birth suite, leaving the postnatal area with less experienced midwives.

Some respondents were very positive about models that provided for increased continuity of care by a system of staff rotation, and negative toward core staff, or staff who were reluctant to work in all areas. Two key informants said that they had tried to introduce rotations or team care in their unit to enhance continuity but it just did not work in their particular situation. The issue of postnatal staff often being moved to other areas that are short of staff (sometimes just after a midwife has met the women and told then she will be caring for them) also impacts on continuity in postnatal wards:

Continuity of care falls down...in the postnatal area because of movement out of the area to fill in gaps elsewhere, so it's difficult [and] team midwives who tend to get pulled out and moved elsewhere find it really hard because they enjoy doing postnatal [and providing continuity] (KI-1048-2, tertiary)

#### Staff-patient ratios

Eight key informants made comments regarding the inadequacy of the staff/patient ratios, particularly in relation to the overnight staff ratios, where it is one midwife to eight women (plus their babies). Comments that captured these views included:

It's interesting with the ratios too because the ratios through the day compared [with those] overnight...when we are looking at our breastfeeding stats it's overnight when the mums need the most support when they're exhausted and the babies are not feeding [and] they tend to be wanting as much support as possible (KI-1023-1, regional)

Night duty can be as busy as day duty, not all the time, but it can be, and we have 1 to 8...it can be almost horrific (KI-1046-1, tertiary)

#### Staff distress related to adequacy of staffing

An emerging theme from key informant interviews was the negative impact on staff related to perceived inadequacy of staffing. Thirteen key informants (34%) made comments about this, with descriptive words including "stressful", "demanding", "difficult", "pressured" and "frustrating".

I've had...patients communicating [a] feeling of neglect...and it would tend to be when staff are too busy to actually give the care they would like to give... [it is a] time factor (KI-1035-4, metropolitan non-tertiary)

...if it does get really busy and you are unable to get extra midwives to work [it] gets a bit stressful...to try and concentrate on someone in labour and trying to help someone who's got breastfeeding problems in the ward (KI-1036-1, rural)

Morale is very low at the moment because [staff] know they are not delivering the care they want to give...things are having to be missed...they're having to prioritise...there are tasks they're not getting to, and it's affecting morale (KI-1059-1, metropolitan non-tertiary)

These situations were heightened in areas where staff had to care for new mothers and babies as well as other patients. Other impacts included staff feeling troubled because they had a sense that they had to function in a very reactive way due to the busy environment. At times this meant that the women who were most assertive were the ones likely to get the care, whereas those who were not might miss out somewhat:

...some people just know how to ask for help better than others, and sometimes you just have to say 'I'm sorry but I can't' and that's very difficult too...(KI-1020-3, regional)

There will always be a patient ...that will [get] more care than ... other people who probably require the care but don't speak up...because they are too timid or unable to communicate their needs (KI-1020-4, regional)

### Recruitment and retention of midwives

Ninety-two percent (61/66) of survey respondents commented on a question regarding issues with the recruitment and retention of midwives. Of these, 34% (21/61) reported no problems recruiting or retaining midwives. The remaining 66% (40/61) discussed issues with recruitment and retention of midwives, with recruitment being the greater problem. Rural hospitals reported the most difficulties: 62% (24/39) of rural hospitals and 56% (5/9) of regional hospitals reported recruitment of midwives as a problem. No metropolitan non-tertiary and one tertiary hospital reported recruitment as an issue.

Of those that reported issues with recruitment and/or retention, there were four key areas which had an impact: being in a rural location; having mixed wards; the ability of midwives to maintain their skills; and the age of the midwives.

#### Being in a rural location

Many rural respondents commented that being located in a rural area was a significant contributing factor to staff recruitment difficulties:

As with many rural hospitals it's difficult to attract new midwives unless they already have ties to this area. Retention does not appear to be an issue here (survey-1041, rural)

Recruitment usually an ongoing issue due to the hospital being in a rural area (survey-1028, rural)

The issue of recruiting and retaining midwives in rural areas was also reported to be related to employment opportunities for the midwives' partners:

Geographical isolation [is an issue, with] lack of employment opportunities for spouses (survey-1074, regional)

Retention often depends on their partner's job too (survey-1047, regional)

#### Mixed wards

Having mixed wards may not be an attractive option for midwives wanting to practice solely in midwifery. Again, this is more likely to be the case in rural areas, where mixed wards are more common:

It is very difficult in the rural areas to recruit/retain midwifery staff who spend 90% of their time doing general ward work, mainly geriatrics. Birthing numbers are small and midwifery work satisfaction is minimal (survey-1024, rural)

Difficult to recruit young single people to country areas – workload will include acute medical/surgical patients plus [accident and emergency] – small amount of midwifery work (survey-1039, rural)

#### Midwifery skill maintenance

Rural midwives in particular may have difficulty in maintaining their skill levels due to low birth numbers, as well as the 'low risk' status of many of the women for whom they provide care. There may often be limited opportunities to maintain the skills required to care for emergencies or women of higher 'risk'.

Recruitment is extremely difficult as not many midwives want to work at a small country hospital due to the lack of midwifery contact hours they get. A lot of midwives are also worried about losing their skills (survey-1015, rural)

We have a small number of deliveries and you are expected to care for general patients as well. Difficult to maintain skills (survey-1049, rural)

#### The age of the midwives

Survey respondents reported that the ageing midwifery workforce (again particularly in rural areas, although this is a state-wide phenomenon) compounded problems of staff retention. There was a sense that many of the older midwives may not work much longer, and that it would be difficult to replace them:

The average age of our midwives is 45+. Midwives are tired. Many have cut down their hours (survey- 1019, regional)

[The] main issue with midwives is the age range of our present staff with a large percentage over 50 years of age (survey-1026, rural)

### Staffing solutions

Key informants were asked about their vision of postnatal care in an ideal world, and of the 36 who responded to this question, 13 (36%) commented on staffing, saying that improved and/or more flexible staffing would be ideal. The main focus of these comments was a desire for midwives to have more time with individual women to improve outcomes like establishing breastfeeding and patient satisfaction with care. A ratio of one midwife to three or four postnatal women was suggested by six respondents.

...one staff member to four patients would be ideal, and four 'postnates' because you've got babies, and that way you could spend more time with them and educating them, helping them with their baby (KI-1020-2, regional)

More staff, to women ... more importance placed on that period of time by management so that they would ... staff it appropriately, more importance placed on it, recognising it (KI-1035-3, metropolitan non-tertiary)

#### Strategies for recruitment and retention

Respondents suggested a number of strategies to address problems with staff recruitment and retention including financing of registered nurses to undertake a Postgraduate Diploma in Midwifery and providing clinical placements for students, in the hope that this would attract these people to work there when they had finished:

Hospital management have financed the training of 5 Div 1 nurses in midwifery in the past 5 years – this has worked very well (survey-1039, rural)

In recent years providing clinical placement for student midwives has provided us with a regular supply of midwives (survey-1057, regional)

Five survey respondents commented that a new or modified model of maternity care (such as caseload or team midwifery) might attract and retain midwives and help ensure the sustainability of their service:

I am proposing yet again a team or caseload model of care ... for sustainability of our service because staffing is a major problem and [we] cannot maintain the current system for much longer (survey-1024, rural)

## Discussion

Staffing of hospital postnatal units in Victoria is a significant and complex issue, and was highlighted in both the survey and key informant interviews as having a major impact on the provision of quality postnatal care. Many respondents had a great deal to say about staff numbers and ratios, as well as skill mix, staff mix and staff recruitment and retention. In the majority of cases the staffing of postnatal wards is provided by a variety of staff (mostly midwives), employed in a combination of ways. The 'Nurses (Victorian health sector) multiple business agreement' states that "*where appropriate, the ratios in [the list of smaller hospitals both rural and metropolitan] may be reached with a mix of Registered Nurses Divisions 1 and 2*" [11]. Mothercraft nurses are not mentioned. Other key issues raised in our review were the prioritisation of other areas over postnatal, and the various factors impacting on organisations' ability to provide continuity of care.

Many providers considered that staff/patient ratios in the area of postnatal care are inadequate. Two key factors contributing to this are that the mother and baby are not treated as separate entities; and that the proportion of mothers and babies with increased needs has increased greatly in the last decade. For example, the most recent Victorian state-wide figures show that 29.5% of births are by caesarean section [17], thus actually meeting the surgical staffing requirements for a staff/patient ratio of one to four (plus a person in charge) on a morning and afternoon shift [11]. This also means that there are fewer mothers able to contribute to caring for their babies. There are more babies of higher acuity being cared for on postnatal wards rather than in special care nurseries, such as more premature infants, and those requiring intravenous antibiotics. Staffing requirements in this climate of increased numbers of new mothers with post-surgical needs as well as increased numbers of higher needs babies should be reviewed and monitored by the three key bodies, the ANF, ACMI and DHS. Alternate ways of considering staffing in the provision of maternity services could also be considered, and there are various patient dependency packages in use. In NSW (another state of Australia), a new package is being piloted and introduced [18] as a statewide strategic workforce planning method which is individualised to each institution. Retrospective data is collected over several months to enable accurate assessment of patient acuity and case mix, and hence staffing needs.[19]

The majority of hospitals reported a mix of staff providing postnatal care, and only 43% of hospitals report having just midwives (i.e. no non-midwives) providing hospital postnatal care. None of the other three staff categories (Division 2 nurses; Division 1 nurses not endorsed as midwives; or mothercraft nurses) is qualified to provide the full range of care needed by postnatal women and their babies. As well as leading to increased staffing requirements, there may be fragmentation of care. For example a mothercraft nurse may teach breastfeeding and baby care, with a midwife required to undertake other aspects of care such as giving intravenous antibiotics (to the mother and/or the baby), checking the involution of the fundus, performing the newborn screening test or administering a vaccine. As well as this potential loss of continuity of care, staff satisfaction may be less as result of this type of approach, leading to decreased staff retention. Already in rural Victoria there are significant workforce issues in maternity services, with a shortage of midwives as well as obstetricians [20]. It is imperative to listen to staff views in this climate of staff shortages, to optimise staff retention and ensure care provision for women. It is also important that health services explore innovative and flexible approaches to staff recruitment and retention.

Many of the issues arising when discussing staffing in relation to postnatal care are interconnected, for example the way in which care is structured and organised may impact on the number and type of staff required. Similarly, organisational decisions about what care should be provided during a woman's postnatal stay will impact on staffing requirements, including factors such as average length of stay, rooming in, and timing and type of routine observations of mother and baby. These are all major factors, and will be reported elsewhere. A number of key issues around postnatal care identified in this review were related to rural location, and although the SRM 2000 findings that women in rural Victoria were more likely to rate their care as 'very good' compared to women in metropolitan areas [21], the difference was only 55% rating their care as 'very good' in rural areas compared with 50% in metropolitan. In rural areas midwifery recruitment and retention were highlighted as issues, as were midwifery skill maintenance, and staffing adequacy and skill mix. Possible explanations include the increased choice women have regarding length of stay in many rural hospitals, or that local community-based care in itself enhances the woman's experience of postnatal care.

We were unable to locate any literature reporting on studies that looked directly at the organisation and staffing of postnatal care in the same way we have in the PinC review. Dykes found in her ethnographic study of midwives and breastfeeding women that there needs to be a reorganisation of postnatal hospital care, and that staffing of postnatal wards should be reviewed to ensure that midwives have sufficient time to provide care [22]. It is likely that the capacity for staff to be less rushed, more sensitive to women's needs and more able to provide helpful advice and support is related at least in part to staffing adequacy. Limited staff time is likely to equate to limited time available to spend with women.

The PinC review used two methods of data collection: a survey and semi-structured interviews. The key informants were chosen in a purposive way to reflect diverse settings and perspectives but still may not have captured the full range of care providers' views on postnatal care. Additionally, we would ideally continue data collection to further explore the issues that arose in the analyses, to enable further consideration of 'where to from here?'. The current review included only the public sector, but we know that women in private hospitals rate their postnatal care more favourably than those in public hospitals; we are currently undertaking a review of postnatal care provision in private hospitals. Another possible issue is that while the majority of births in the state take place in metropolitan Melbourne, 53/66 (80%) survey respondents were from rural Victoria, which is consistent with the proportion of maternity services that are based in those areas. The rural providers are therefore arguably over-represented in this survey in terms of the proportion of births that take place in the rural sector.

The PinC review raised a number of issues where further research and/or policy changes would be helpful in order to consider possibilities for staffing in postnatal care. Recruitment and retention of staff to provide postnatal care in rural Victoria in particular is a key area where policy changes may make a difference, and this is currently being addressed in part by the Victorian Rural Maternity Initiative [23]. Areas to explore could include: the further development of models of midwifery care that promote continuity of care in the early postnatal period, with options for rural midwives to provide a continuum of postnatal care in both the hospital and home setting; minimising the current practice of accommodating new mothers and their infants with non-maternity patients and ensuring that postnatal care is provided by midwives; encouraging student placements in rural hospitals with initiatives such as financial support for Division 1 nurses to undertake midwifery programs; and the promotion of skill enhancement opportunities such as midwife exchange programs between metropolitan, regional and rural sectors, hospital-sponsored visiting academics, researchers, and clinicians, and use of distance learning opportunities/learning packages.

## Conclusion

Staffing in postnatal wards is a complex issue that needs to be addressed by policy makers. Staff/patient ratios and recruitment of midwives across the state, particularly in rural areas, are the two areas that appear to have the greatest negative impact on staffing adequacy and therefore potentially on the provision of quality care. Future research on postnatal care provision should include consideration of any impact on staff and staffing. The review findings, together with other research on women's satisfaction with care, will help inform new approaches to the provision of care aimed at improving women's and caregivers' satisfaction with the early postnatal experience, and maternal and infant health outcomes.

## Competing interests

The author(s) declare that they have no competing interests.

## Authors' contributions

Della Forster (DF), Helen McLachlan (HMc), Jane Yelland (JY), Jo Rayner (JR), Judith Lumley (JL) and Mary-Ann Davey (MAD)

Chief investigators/overall project responsibility: DF, HMc, JY

Study conception: DF, HMc, JY, JL

Study design: DF, HMc, JY

Grant applications: DF, HMc, JY, JL

Questionnaire development, piloting and completion: DF, HMc, JY, JR, MAD, JL

Coordination and implementation of project: DF, HMc, JY, JR

Development of data collection process: DF, HMc, JY, JR

Data collection and management: DF, HMc, JY, JR, MAD

Data analysis and interpretation: DF, HMc, JY, JR, MAD

Drafted manuscript: DF

All authors read and approved final manuscript.

## Pre-publication history

The pre-publication history for this paper can be accessed here:



## Figures and Tables

**Table 1:** Participating hospitals by number of births (2002)

No. of births in 2002	No. of hospitals	% of hospitals (n = 66)
No births (postnatal care only)	6	*9.1*
< 100	21	*31.8*
100–499	21	*31.8*
500–999	6	*9.1*
1000–1,499	4	*6.1*
>= 1,500	8	*12.1*

**Table 2:** Staff other than midwives providing postnatal care in participating hospitals by hospital category

**Profession**	**Rural **		**Regional **		**Metropolitan (non-tertiary) **		**Tertiary **		**Totals **	
	(n = 28)		(n = 10)		(n = 8)		(n = 3)		(n = 49)	
	n*	*%*	n*	*%*	n*	*%*	n*	*%*	n	*%*
Mothercraft nurses	2	*7.1*	3	*30.0*	3	*37.5*	2	*66.7*	10	*15.0*
Division 2 nurses	9	*32.1*	6	*60.0*	2	*25.0*	1	*33.3*	18	*27.0*
Registered nurses	5	*17.9*	1	*10.0*	0	*-*	0	*-*	6	*9.0*
										
**Hospitals where only midwives provide postnatal care**	16	*57.1*	2	*20.0*	3	*37.5*	0	*-*	21	*42.9*

* The number of hospitals in each category who reported employing care givers of each type
